# Is the intentionality of mind wandering associated with the combined dimensions of temporal orientation and emotional valence?

**DOI:** 10.1186/s40359-025-03821-7

**Published:** 2026-02-21

**Authors:** Siqing Guan, Toru Takahashi, Hiroaki Kumano

**Affiliations:** 1 Research Area of Clinical Psychology, Graduate School of Human Sciences, Waseda University, Tokorozawa, Japan; 2 Japan Society for the Promotion of Science, Tokyo, Japan; 3 Laureate Institute for Brain Research, Tulsa, OK USA; 4 Faculty of Human Sciences, Waseda University, Tokorozawa, Japan; 5 Graduate School of Human Sciences, Waseda University, 2-579-15, Mikajima, Tokorozawa, Saitama 359-1164 Japan

**Keywords:** Mind wandering, Intentionality, Temporal orientation, Emotional valence

## Abstract

Mind wandering (MW) varies in its functional consequences depending on both its intentionality (intentional vs. unintentional) and the content of thought. While previous studies have examined these dimensions separately, little is known about how combinations of content features—such as temporal orientation and emotional valence—are shaped by intentionality. This study investigated whether intentional and unintentional MW differ in content when temporal orientation (past vs. future) and emotional valence (positive, neutral, negative) are considered in combination. Participants reported the content of their MW episodes, which were categorized accordingly. The results showed that unintentional MW was more frequently associated with neutral or negative past-oriented content, whereas intentional MW was more often linked to positive or negative future-oriented content. These findings suggest that the effects of MW content vary depending on intentionality, particularly when multiple content dimensions are considered together. This study contributes to a deeper understanding of the functional heterogeneity of MW by highlighting the interaction between intentionality and thought content.

**Supplementary Information:**

The online version contains supplementary material available at 10.1186/s40359-025-03821-7.

## Introduction

Mind wandering (MW) refers to shifts of attention away from an ongoing task toward internally generated, task-unrelated thoughts [1]. Recent estimates suggest that individuals spend approximately 10–50% of their waking hours engaged in MW [2, 3]. Although ubiquitous, MW demonstrates considerable functional heterogeneity; it can yield beneficial outcomes, such as enhanced creativity and future planning, as well as detrimental consequences, including impaired task performance and increased negative emotionality [4].

### Content dimensions of Mind wandering

The content regulation hypothesis [5] posits that the functional consequences of MW depend largely on its phenomenological content—especially temporal orientation (past vs. future) and emotional valence (positive, neutral, or negative). Temporal orientation distinguishes MW content that disengages attention from the present moment by focusing on the past (e.g., memory consolidation) or the future (e.g., goal planning) [6, 7]. Emotional valence further characterizes MW content as either emotionally positive and potentially beneficial for well-being, or emotionally negative and potentially detrimental — with the latter often linked to mood disorders [8, 9]. Notably, the combination of these dimensions—rather than each alone—may have unique implications. Future-oriented and positively valenced MW may support optimism and adaptive planning [8, 10]. In contrast, negatively valenced MW has been consistently associated with maladaptive cognitive outcomes [11]. Although prior research has not consistently demonstrated a direct association between temporal orientation and rumination [12, 13], recent studies that differentiate intentionality have shown that unintentional, future-oriented MW is significantly associated with rumination and worry [11]. These findings suggest that understanding the functional implications of MW requires considering the interaction of multiple dimensions—such as intentionality and content dimensions—rather than focusing on temporal orientation alone.

#### Intentionality in Mind wandering

An equally important but often understudied dimension is intentionality, which distinguishes intentional (voluntary) MW from unintentional (spontaneous) MW. According to conceptual definitions [14, 15], intentional MW involves a top-down, goal-driven shift of attention away from the external environment, typically motivated by internal goals or reasoning. In contrast, unintentional MW arises involuntarily due to failures in executive control and reflects bottom-up processes. These two types of MW differ not only in their initiation mechanisms but also in their functional outcomes: unintentional MW is positively associated with depression, anxiety, and stress, while intentional MW is weakly negatively associated with stress and anxiety and shows no relationship with depression [16].

Importantly, intentionality may also shape the emotional functions of MW. Unintentional MW often overlaps with maladaptive processes such as rumination (repetitive thinking about past negative events) and worry (repetitive anticipation of potential future threats), which are linked to depression and anxiety. In contrast, intentional MW may be more closely aligned with adaptive processes, including nostalgia — a sentimental longing for meaningful past experiences that fosters meaning-making and social connectedness — and mental contrasting — envisioning a desired future while reflecting on present obstacles, which supports emotional preparation and strengthens goal commitment. These distinctions provide a richer framework for understanding how MW supports or undermines emotion regulation.

#### Theoretical perspectives on Mind wandering

Traditionally, MW has been conceptualized as a unitary phenomenon, most notably through two influential frameworks: the decoupling hypothesis and the executive failure hypothesis. The decoupling hypothesis [17] proposes that MW arises when attentional resources are redirected from external stimuli to internally generated thoughts, resulting in perceptual disengagement. In contrast, the executive failure hypothesis [18] posits that MW stems from lapses in executive control, allowing task-unrelated thoughts to intrude involuntarily.

Importantly, early models implicitly treated MW as an involuntary process and did not explicitly consider the role of intentionality—that is, whether MW arises deliberately or spontaneously. Although MW has often been characterized as spontaneous and difficult to control, especially within theoretical accounts from the 2000 s that emphasized unintentional forms of MW [15], earlier research had already recognized that individuals sometimes voluntarily disengage from external tasks to engage in internal thoughts [14, 19, 20]. In line with this broader perspective, the present study explicitly treats intentionality as a central dimension of MW. Building on this foundation, recent theoretical developments—particularly those by Seli and his colleagues [15, 21]—have further challenged the assumption that MW is necessarily unintentional. Their work introduced a now widely adopted distinction between intentional MW, which is deliberately initiated and maintained, and unintentional MW, which arises spontaneously without intention. This differentiation in control styles has garnered substantial empirical and theoretical support, leading to a reconceptualization of MW as encompassing both controlled and uncontrolled cognitive states.

Empirical findings support this revised view. Behavioral studies have shown that intentional MW is more likely to occur under low cognitive load, when mental resources are available for internal thought, whereas unintentional MW becomes more frequent under high-load conditions [22]. Neuroimaging evidence further reinforces this distinction: intentional MW is associated with increased connectivity between the default mode network (DMN) and the frontoparietal control network (FPCN), indicating top-down regulation, while unintentional MW is linked to increased intra-DMN connectivity, suggesting reduced executive oversight [23]. Taken together, these findings suggest that intentional MW may be best understood through the lens of the decoupling hypothesis—reflecting deliberate disengagement from the external environment—whereas unintentional MW aligns more closely with the executive failure hypothesis, representing spontaneous attentional lapses due to diminished cognitive control.

#### The present study

In light of recent theoretical advances and growing empirical evidence, a more integrated investigation of how intentionality shapes the emotional and temporal characteristics of MW is both timely and necessary. Although prior studies have examined MW’s content dimensions and intentionality in isolation, relatively few have explored how specific combinations of temporal orientation and emotional valence systematically differ depending on whether MW is intentional or unintentional [24].

This gap is significant, as distinct intentionality–content profiles may have differential implications for emotional and cognitive functioning. For instance, previous research suggests that unintentional MW is commonly characterized by negatively valenced, past-oriented thoughts, which are associated with depressive rumination and anxiety [25]. In contrast, intentional MW—even when involving negative content—may support adaptive processes such as emotional preparation, nostalgia, mental contrasting, goal-directed planning [26, 27]. Moreover, the absence of positively valenced, future-oriented thought—rather than the presence of negative content—has been identified as a stronger predictor of depression and suicidality [28, 29].

Building on these findings, the present study integrates the content regulation hypothesis [5], the decoupling hypothesis [17], and the executive failure hypothesis [18] to examine how intentionality influences the co-occurrence of temporal and emotional content in MW. This approach aligns with recent frameworks that conceptualize intentional and unintentional MW as qualitatively distinct phenomena, each supported by different cognitive control mechanisms [15, 22]. Given these theoretical and empirical distinctions, we hypothesized that the emotional and temporal features of MW content would systematically vary as a function of intentionality.

Accordingly, we tested the following hypotheses:

- Hypothesis 1: Compared to intentional MW, unintentional MW is more likely to involve negatively valenced, past-oriented content.
- Hypothesis 2: Compared to intentional MW, unintentional MW is less likely to involve positively valenced, future-oriented content.

## Methods

### Participants

Data from 30 participants (19 women, 11 men; *M* age = 23.5 years, *SD* = 4.5) were included in the final analyses. The target sample size was determined based on simulation studies by [30], which demonstrated that fixed-effect estimates in multilevel logistic regression remain unbiased when there are at least 30 level-2 units (i.e., participants). To ensure sufficient within-person observations, each participant completed 20 thought-sampling probes, consistent with prior experience-sampling studies of MW [22, 31].

A priori power analysis was conducted using a simulation-based web application for multilevel logistic regression [32]. With α set at 0.05 and β at 0.20 (80% power), a sample size of 30 participants with 20 level 1 observations per person was considered sufficient to detect small-to-moderate fixed effects with adequate power. Each category contained more than 20 valid MW events, thereby exceeding the threshold of 10 events per predictor variable recommended for stable logistic regression estimation [33].

Participants were healthy undergraduate and graduate students recruited from a local Japanese university via an online student job portal and campus advertisements. A total of 31 individuals completed the study and received 1,500 yen as compensation. One participant was excluded due to a task administration error. The final sample thus comprised 30 participants.

### Measures

#### Sequential sustained attention to response task (Sequential SART)

Participants completed a Sequential Sustained Attention to Response Task (Sequential SART), during which thought probes were intermittently presented to assess the presence and content of MW. Thought sampling during sequential SART is a well-established method for capturing MW [22, 31].

This study employed a sequential version of the SART designed to reduce cognitive load and allow for the occurrence of both intentional and unintentional MW [22, 31]. This design facilitates natural MW while maintaining sufficient attentional demands. The task was programmed in PsychoPy2 following procedures outlined in previous studies. On each trial, a digit (1–9) was presented for 250 ms, followed by an “X” mask for 900 ms (trial duration: 1,150 ms). Digits appeared sequentially (1–9) and varied randomly in one of five font sizes (48–120 pt, Courier New). In each nine-trial block, four font sizes were shown twice, and one once. Participants pressed the spacebar for every digit except 3, which required response inhibition. After 18 practice trials (2 blocks), participants completed 900 experimental trials (100 blocks).

### Thought probes

MW was assessed using a variant of the experience-sampling method, where participants periodically responded to thought probes during the task [22]. Participants indicated whether they were on-task or experiencing intentional or unintentional MW immediately before the probe. Intentional MW was defined as task-unrelated thoughts generated deliberately; unintentional MW as involuntary, task-unrelated thoughts [22, 31].

Each probe also assessed two MW content dimensions [24, 34, 35]: temporal orientation (past vs. future) and emotional valence (positive, neutral, negative). These were evaluated both independently and in combination to allow comprehensive analysis. Probes were presented during 900 experimental trials, with 20 probes distributed randomly across Blocks 2 to 98 (no more than one per block). The probe questions, presented in Japanese, were as follows:

1. “Which of the following best describes your mental state immediately before this screen appeared?”
  (1) On task, (2) Intentional MW, (3) Unintentional MW.
2. “Was your thought content related to the past or the future?”
  (1) Future events, (2) Past events.
3. “What was the emotional tone of your thoughts?”
  (1) Positive, (2) Neutral, (3) Negative.

All questions were administered regardless of the response to Question 1, allowing for complete data collection. MW content data were scored only for intentional or unintentional MW responses. The reliability and validity of this probe method have been supported in previous research [36].

### Procedure

Before beginning the experiment, participants completed a screening questionnaire to ensure they had not taken medication in the past 24 h, consumed alcohol in the past 12 h, or consumed caffeine in the past 6 h.

Written informed consent was obtained from all participants, and all procedures were approved by the Ethics Committee for Research on Human Subjects at Waseda University.

After providing consent, participants received standardized instructions and completed a brief familiarization session with the SART task. Prior to the main task, they were informed that the session would last approximately 25–30 min, although the exact number of thought probes was not disclosed.

Instructions for the thought-probe method were adapted from Seli et al. [22], translated into Japanese, and explained both orally and in writing. Participants were presented with the following definitions:

- **ON TASK**: Just before the screen appeared, you were focused on completing the task and were not thinking about anything unrelated to it. This includes thoughts about your performance, the digits, or your responses.
- **MW**: Just before the screen appeared, you were thinking about something completely unrelated to the task.
  - **Unintentional MW**: You unintentionally drifted to task-unrelated thoughts, despite your best intentions to stay focused.
  - **Intentional MW**: You deliberately decided to think about things unrelated to the task.

These instructions were provided to ensure participants clearly understood the distinctions between MW types and to enhance the transparency and reproducibility of the experience sampling procedure.

### Data analysis

Descriptive statistics for performance on the sequential SART task were first computed to characterize participants’ behavioral responses. Subsequently, a series of multilevel binary logistic regression analyses was conducted to examine whether the temporal orientation and emotional valence of MW content varied as a function of intentionality (i.e., intentional vs. unintentional MW).

Each binary outcome variable represented a specific content dimension (e.g., negative past-oriented content), and was coded as 1 when the given MW episode matched that content type, and as 0 when it did not. In other words, each model predicted the probability of a specific MW type occurring versus all other types. MW intentionality (0 = intentional, 1 = unintentional) was entered as a fixed-effect predictor, and random intercepts were included for participants to account for repeated measures nested within individuals (Level 1: MW episodes; Level 2: participants). All Level 1 predictors were person-mean centered to isolate within-person variability from between-person differences, in line with the primary aim of examining moment-to-moment fluctuations in MW content.

For each model, intraclass correlation coefficients (ICC) were computed to assess the proportion of variance attributable to between-person differences and to justify the multilevel approach. ICC ranged from 0.015 (positive past-oriented content) to 0.222 (neutral content), indicating variable but non-negligible between-person clustering.

The significance threshold was set at α = 0.05 (two-tailed) for all analyses. In addition to reporting odds ratios and 95% confidence intervals, the natural logarithm of the odds ratio (log[OR]) was calculated to clarify both the magnitude and direction of each effect. The log-transformation allows effect sizes to be interpreted on a symmetrical scale, with values around 0 indicating no effect, positive values indicating greater likelihood in unintentional MW, and negative values indicating greater likelihood in intentional MW. This approach facilitates the comparison of effect sizes across categories and is especially informative in logistic regression [37].

## Results

### Descriptive statistics

On average, participants responded to go-trials within 246.80 ms (SD = 74.17) and made errors on 21.10% (SD = 10.97) of no-go trials. Of the total responses, 266 task-focused reports were excluded, and 334 MW episodes were retained for further analysis.

To justify the use of multilevel logistic regression, ICC were calculated for each MW content type. Each binary outcome variable was coded as 1 for the presence of a specific content dimension and 0 otherwise. ICC ranged from 0.015 (positive past-oriented content), indicating negligible between-person clustering, to 0.222 (neutral content), suggesting moderate variability across individuals. These values support the use of multilevel modeling to account for both within- and between-person variability. Notably, the ICC for positive past-oriented content was not statistically significant, indicating that this content type may primarily reflect within-person fluctuations (Table 1).

**Table 1:** Frequencies (%), total, and ICC of intentional and unintentional MW by content types

MW Types	*N*	Frequencies	ICC	*p*
Intentional MW	95	27.6%	0.071	0.006
Unintentional MW	239	69.5%		
Past-oriented content	153	44.5%	0.149	< 0.001
Future-oriented content	181	52.6%		
Positive content	73	21.2%	0.206	< 0.001
Neutral content	200	58.1%	0.222	< 0.001
Negative content	61	17.7%	0.119	< 0.001
Positive past-oriented content	21	6.1%	0.015	0.259
Neutral past-oriented content	103	29.9%	0.138	< 0.001
Negative past-oriented content	26	7.6%	0.092	0.001
Positive future-oriented content	52	15.1%	0.180	< 0.001
Neutral future-oriented content	96	27.9%	0.116	< 0.001
Negative future-oriented content	33	9.9%	0.089	0.001

Note:* ICC* Intraclass correlation coefficient calculated for each MW content type, *n.s.* not significant

Intentional and unintentional MW percentages are based on the total number of responses within each category

### Differences in the combination of temporal orientation and emotional valence between intentional and unintentional MW

To examine how MW intentionality associated with phenomenological content, a series of multilevel binary logistic regression models was conducted. Each model predicted the likelihood of reporting a specific MW content type (coded as 1) versus all other types (coded as 0). MW intentionality (0 = intentional, 1 = unintentional) was included as a fixed-effect predictor, with random intercepts for participants to account for repeated measures. All Level 1 predictors were person-mean centered to isolate within-person variability.

As shown in Table 2, unintentional MW was significantly more likely to involve neutral past-oriented content (OR = 1.94, 95% CI [1.05, 3.59], log[OR] = 0.66, *p* =.03) and negative past-oriented content (OR = 4.88, 95% CI [1.08, 21.94], log[OR] = 1.58, *p* =.04). In contrast, unintentional MW was significantly less likely to involve positive future-oriented content (OR = 0.46, 95% CI [0.22, 0.95], log[OR] = − 0.78, *p* =.04) and negative future-oriented content (OR = 0.38, 95% CI [0.17, 0.85], log[OR] = − 0.97, *p* =.02). These log-transformed ORs reflect moderate to large effect sizes, indicating meaningful asymmetries in how MW intentionality shapes content. No significant differences were observed for positive past-oriented or neutral future-oriented content.

**Table 2:**
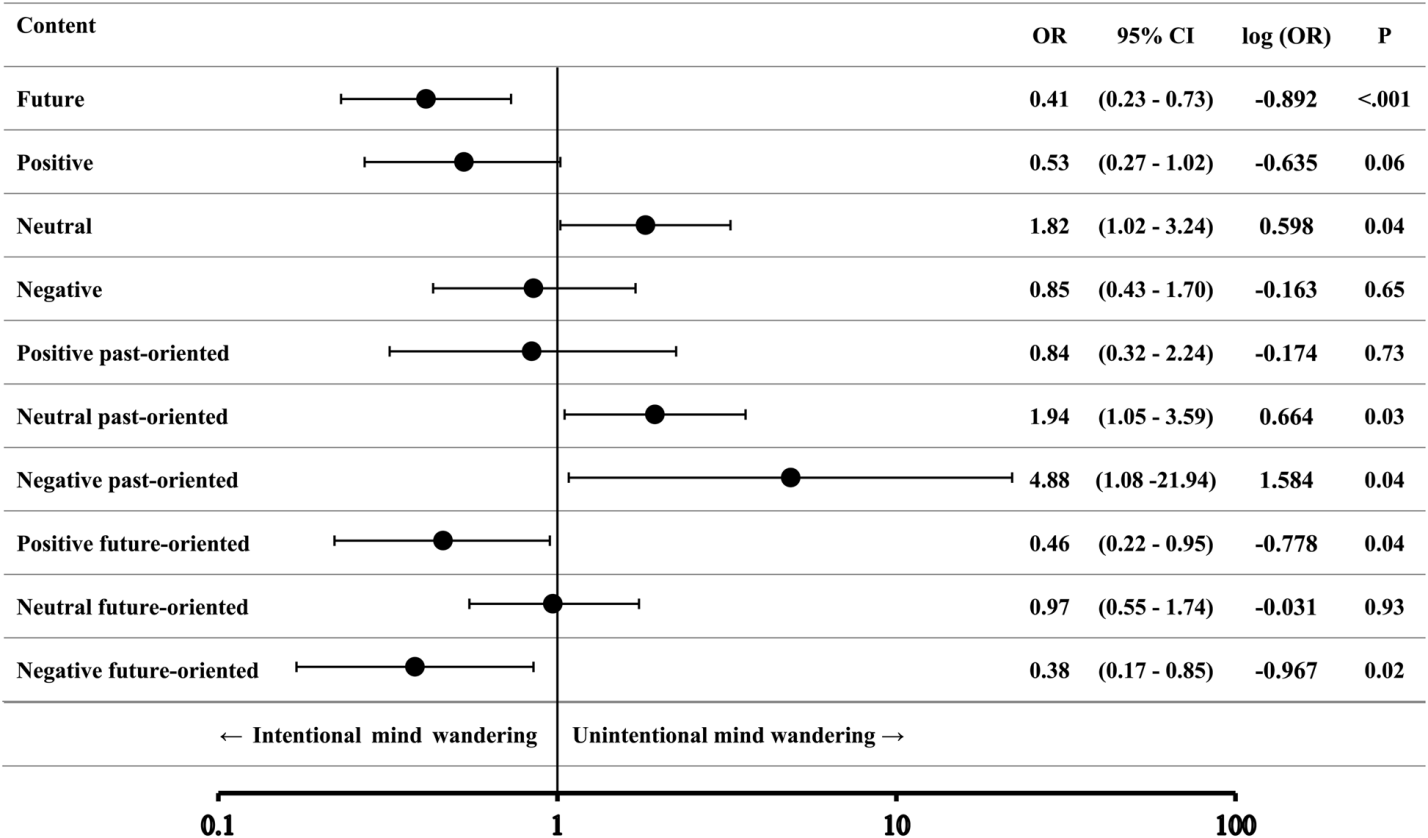
Differences in odds ratio of content between intentional MW and unintentional MW as estimated by multi-level logistic regression

## Discussion

The current study investigated how intentionality (intentional vs. unintentional) is associated with combinations of temporal orientation and emotional valence in MW. Consistent with our hypotheses, unintentional MW was predominantly linked to negatively or neutrally valenced past-oriented content, while intentional MW was more frequently associated with positively or negatively valenced future-oriented content. These findings confirm our hypotheses and clarify how MW intentionality systematically shapes the co-occurrence of temporal orientation and emotional valence. This supports and refines the predictions of the content regulation, executive failure, and decoupling hypotheses, providing a more integrated understanding of MW’s functional heterogeneity.

### The role of intentionality in MW content types

Previous research examining MW intentionality reported no significant differences in emotional valence when considered independently [24]. However, our findings demonstrate clearer distinctions emerge when emotional valence is combined with temporal orientation. Specifically, unintentional MW was more frequently associated with negative or neutral past-oriented content, a pattern consistent with clinical findings linking negative past-oriented cognition to depressive rumination and anxiety [25]. Such findings align with evidence from clinical populations, where the absence of positive future-oriented thought rather than the presence of negative future-oriented content has been strongly correlated with depressive symptoms and suicidality [28, 29]. Although our study utilized a non-clinical sample, these observed patterns suggest potential implications for identifying cognitive vulnerability markers in otherwise healthy individuals.

In contrast, intentional MW showed a clear association with future-oriented content, including negative scenarios. This finding expands prior literature, which typically characterizes future-oriented thought as predominantly positive [26, 38]. Intentional engagement with negatively valenced future-oriented thoughts may reflect adaptive anticipatory processes, such as emotional preparation and proactive coping [27]. Thus, our study provides empirical support for the adaptive function of intentional MW by highlighting strategic emotional ambivalence in future-oriented thoughts, potentially serving self-regulatory and preparatory roles. Nevertheless, distinguishing adaptive anticipatory planning from maladaptive worry remains crucial. Future research should incorporate individual differences (e.g., anxiety sensitivity, intolerance of uncertainty) to further clarify when negative future-oriented MW serves adaptive versus maladaptive functions.

Recent research highlights that unintentional MW is not necessarily maladaptive, nor uniformly past-oriented. In daily life, spontaneous future-oriented thoughts can support goal pursuit, prospective memory, and intention fulfillment by bringing upcoming goals to mind without deliberate effort [39]. This suggests that unintentional MW may have adaptive functions depending on its content and context. In the present study, MW was assessed during a sequential SART, a context in which spontaneous thoughts may more frequently reflect attentional disengagement rather than goal-related future thinking. Therefore, the predominance of negative or past-oriented unintentional MW observed in this study may be shaped by the low cognitive load of the task [22, 31]. Future research is needed to directly compare the phenomenology and functional roles of unintentional MW across different everyday and laboratory contexts.

### Theoretical contributions

The current findings provide empirical support for the theoretical integration of three major frameworks in MW research—the content regulation hypothesis, the executive failure hypothesis, and the decoupling hypothesis. Specifically, our results demonstrate that the intentionality of MW systematically shapes the functional implications of its content, with intentional MW aligning with positive future-oriented thought (supporting the decoupling and content regulation hypotheses), and unintentional MW associated with negative past-oriented thought (supporting the executive failure and content regulation hypotheses). These results help refine and extend each of these frameworks by clarifying their distinct yet complementary explanatory roles.

This study advances the theoretical understanding of MW by integrating three influential frameworks: the content regulation hypothesis [5], the executive failure hypothesis [18], and the decoupling hypothesis [17]. While these frameworks were not directly tested, the present findings offer indirect support and help refine them by clarifying how MW intentionality interacts with temporal and emotional content to shape functional outcomes.

The content regulation hypothesis emphasizes that the consequences of MW depend largely on its content characteristics, such as whether the thoughts are past- or future-oriented and whether they are positively or negatively valenced. In line with this view, our findings show that intentional MW was typically associated with future-oriented content—sometimes even negative—which may support adaptive functions such as goal setting or anticipatory coping. In contrast, unintentional MW was more often associated with negative past-oriented content, suggesting a link to ruminative or dysregulated thought patterns.

These content patterns also map onto complementary cognitive mechanisms proposed by the other two frameworks. According to the executive failure hypothesis, unintentional MW arises from breakdowns in cognitive control, leading to intrusive and maladaptive thoughts. The decoupling hypothesis, by contrast, views intentional MW as a form of strategic disengagement from the external environment, particularly under low-load conditions, to facilitate constructive internal processing. Although we did not directly assess working memory capacity or attentional demands, the observed content differences are broadly consistent with these accounts. Prior behavioral and neuroimaging studies further support this differentiation, showing distinct neural profiles for intentional and unintentional MW [22, 23].

Taken together, these findings suggest that the executive failure and decoupling hypotheses are not mutually exclusive but instead describe distinct but complementary pathways through which MW arises. When considered alongside the content regulation hypothesis, this integrative framework provides a more comprehensive account of MW’s functional heterogeneity—highlighting how both the intentionality of MW and the phenomenological features of its content jointly contribute to its adaptive or maladaptive outcomes.

### Practical implications

Beyond its theoretical contributions, this study offers practical implications for enhancing cognitive and emotional functioning through the adaptive regulation of MW. The findings suggest that intentional MW often involves both positive and negative future-oriented thoughts and may serve beneficial functions such as emotional preparation and goal setting [40, 41]. Such preparation can arise not only from hopeful anticipation but also from recognizing potential obstacles, enabling individuals to mentally rehearse coping strategies before challenges arise. However, future-oriented thinking is not always adaptive; when rigid or negatively valenced, it can lead to worry, rumination, and internalizing symptoms [42, 43]. A balanced strategy like mental contrasting—envisioning a desirable future while acknowledging and addressing potential obstacles—has been shown to enhance goal commitment and motivation, thereby supporting emotionally grounded future planning [44–46]. This framework illustrates how intentional MW can transform both positive and negative future-oriented thoughts into adaptive self-regulatory processes. Accordingly, interventions that foster constructive forms of MW, such as neurofeedback targeting strategic intentional MW, may offer promising avenues for cultivating psychological flexibility, intrinsic motivation, and creativity [47, 48].

In contrast, unintentional MW often involves negative or neutral past-oriented content, reflecting its association with maladaptive cognitive patterns such as rumination, self-referential negativity, and emotional inertia—processes strongly linked to the onset and maintenance of depression and anxiety [11, 43, 49]. Rumination, defined as a repetitive and passive focus on distressing experiences, makes it difficult to disengage from negative content and prolongs emotional distress [43]. Recent evidence further suggests that unintentional MW may initiate a sequential chain, in which negative content triggers rumination and worry, ultimately exacerbating internalizing symptoms of depression and anxiety [11]. These findings illustrate why unintentional MW can undermine emotional regulation, sustain negative mood states, and interfere with goal-directed behavior—underscoring the need for targeted interventions.

These insights clarify why interventions such as mindfulness-based therapy, attention training, and metacognitive approach are promising—not only for reducing unintentional MW, but also for transforming spontaneous thought into adaptive self-reflection may by enhancing awareness and emotional regulation, and attentional flexibility [50–58]. Understanding how intentionality and content interact offers a valuable framework for developing targeted strategies across domains: in clinical settings, to prevent ruminative thought; in education, to support engagement and self-regulation through guided MW; and in workplaces, to enhance creativity and reduce fatigue via structured MW breaks—ultimately promoting psychological resilience and well-being across contexts.

### Limitations and future directions

While the present study contributes to a more nuanced understanding of the functional heterogeneity of MW by integrating intentionality with temporal orientation and emotional valence, several limitations warrant consideration and suggest directions for future research.

First, the cross-sectional design limits our ability to make causal inferences about how MW intentionality influences the co-occurrence of temporal and emotional content. To address this, future studies should employ experience-sampling methods or longitudinal designs, ideally incorporating physiological and behavioral markers (e.g., pupil dilation, heart rate variability, reaction time) to capture the dynamic interplay between intentionality and content in real-time MW episodes [59–62]. In addition, because the present study was not preregistered, future research should aim to enhance transparency and reproducibility through preregistration and independent replication using comparable measurement protocols.

Second, while this study focused on overall group-level patterns, we did not account for potential individual differences or contextual moderators (e.g., trait mindfulness, task difficulty, emotion regulation strategies) that may influence the relationship between MW intentionality and content [63, 64]. Future research should investigate these moderators to better understand “who engages in which types of MW, and under what conditions”, which would inform the development of personalized intervention strategies.

Third, the study sample comprised Japanese university students, which limits the generalizability of the findings. While the intensive repeated-measures design provided sufficient power for detecting within-person effects, the relatively small and demographically homogeneous sample restricts external validity. Future studies should include larger and more diverse populations—including clinical samples (e.g., individuals with depressive or anxiety symptoms), cross-cultural comparisons, and lifespan perspectives—to examine how MW profiles and their functional implications may vary across different sociocultural or developmental contexts.

Fourth, this study relied on self-reported ratings to assess the intentionality, temporal orientation, and emotional valence of MW episodes. While self-reports offer valuable first-person insights into subjective experience, they are susceptible to biases such as limited introspective access, and demand characteristics. Future research could benefit from complementing self-reported measures with objective indices, including eye-tracking and other neurophysiological markers, to triangulate the dynamic properties of MW in real time [65–67].

Lastly, while we focused on general patterns of association, future studies should also examine how real-world outcomes—such as academic performance, emotional resilience, or stress recovery—are linked to specific MW profiles. Such research could directly inform interventions tailored to individual cognitive styles.

By addressing these limitations, future research can further clarify how the interaction between intentionality and content dimensions contributes to adaptive or maladaptive cognitive functioning, supporting both theoretical refinement and applied translation of MW research.

## Conclusions

This study underscores that the intentionality of MW plays a pivotal role in shaping the combinations of temporal orientation and emotional valence, revealing distinct functional patterns and offering a more nuanced understanding of MW’s adaptive and maladaptive tendencies. Specifically, intentional MW tended to involve future-oriented content, which may support potentially adaptive functions—even when such content is negatively valenced. In contrast, unintentional MW was more frequently associated with negative past-oriented content, which might indicate cognitive vulnerabilities in certain contexts.

Integrating MW’s intentionality with its phenomenological dimensions offers valuable theoretical insights into how intentionality shapes the emotional and temporal qualities of MW. These findings also carry tentative implications for intervention strategies—such as cognitive control training, mindfulness practices, and metacognitive approaches—that aim to cultivate more adaptive forms of MW while reducing potentially maladaptive patterns.

## Supplementary Information

Supplementary Material 1.

## Data Availability

The dataset analyzed during the current study is available from the corresponding author upon reasonable request.

## Abbreviations

DMN: Default Mode Network
FPCN: Frontoparietal Control Network
ICC: Intraclass Correlation Coefficient
MW: Mind Wandering
OR: Odds Ratio
SART: Sustained Attention to Response Task

## Declarations

### Openness and transparency statement

This study was not pre-registered. All analysis scripts, post-hoc power simulations, and task materials (including Japanese originals and English translations of the instructions) are openly available at the Supplementary Materials. AI-assisted tools (ChatGPT, GPT-5; and Grammarly) were used for proofreading and wording refinement only; no content generation, data analysis, or interpretation was conducted by AI. All final conceptual, analytical, and interpretative decisions were made by the authors.
