# Supplementary material for: Is the intentionality of mind wandering associated with the combined dimensions of temporal orientation and emotional valence?
Source: BMC Psychol. 2026 Feb 21;14:428. doi: 10.1186/s40359-025-03821-7 (PMC13032290; doi:10.1186/s40359-025-03821-7)
Supplement: Supplementary file 1 — Supplementary Material 1. [file 40359_2025_3821_MOESM1_ESM.docx]

**Supplementary Materials**

**Analysis Scripts with R code**

Each binary outcome variable represented a specific content dimension (e.g., negative past-oriented content) and was coded as 1 when the given MW episode matched that content type and 0 otherwise. MW intentionality (0 = intentional, 1 = unintentional) was entered as a fixed-effect predictor, and random intercepts were included for participants to account for repeated measures (Level 1: MW episodes; Level 2: participants). All Level 1 predictors were person-mean centered to isolate within-person variability from between-person differences.

----------------------------------------------------------

## Required packages

library(dplyr)

library(lme4)

## ----------------------------------------------------------

## 0. Data structure (example)

## ----------------------------------------------------------

## mw_data:

## id : Participant ID (Level 2)

## MW_intent : MW intentionality (0 = intentional, 1 = unintentional)

## neg_past : Binary outcome (1 = negative past-oriented MW, 0 = otherwise)

##

## If you have additional Level 1 predictors (e.g., affect variables),

## add their names to the vector below.

level1_vars <- c("MW_intent") # Add more Level 1 predictors if needed

## ----------------------------------------------------------

## 1. Person-mean centering of Level 1 predictors

## ----------------------------------------------------------

mw_data <- mw_data %>%

group_by(id) %>%

mutate(

across(

all_of(level1_vars),

~ .x - mean(.x, na.rm = TRUE), # person-mean centering

.names = "{.col}_c"

)

) %>%

ungroup()

## ----------------------------------------------------------

## 2. Multilevel logistic regression for a specific content type

## Example outcome: negative past-oriented content (neg_past)

## Each model predicts: "this content type vs all other MW types"

## ----------------------------------------------------------

model_neg_past <- glmer(

neg_past ~ MW_intent_c + # fixed effect: person-mean centered intentionality

(1 | id), # random intercepts for participants

data = mw_data,

family = binomial(link = "logit")

)

summary(model_neg_past)

**Post-hoc Power Considerations**

Consistent with statistical recommendations (Gelman & Carlin, 2014), post-hoc power analysis was not conducted in the main manuscript because observed power is a direct transformation of the obtained p-value and therefore does not provide additional information regarding sample adequacy. In the main text, we instead report effect sizes (odds ratios), 95% confidence intervals, and p-values for all fixed effects, which represent the recommended indices of estimate precision for multilevel logistic models.

However, for completeness and to facilitate transparency, we provide *approximate* post-hoc power estimates in this supplementary material. These values are reported solely to assist readers who may wish to consider an additional sensitivity index and should be interpreted cautiously, given that post-hoc power does not offer inferential insights beyond those already conveyed by the confidence intervals.

Post-hoc power analyses were conducted using the *NormalIndPower* function from Python’s statsmodels package (version 0.14.0), based on the observed odds ratios, the empirical sample sizes (N = 95 for intentional MW; N = 239 for unintentional MW), and an α level of .05. Because the base rates of intentional and unintentional MW episodes show considerable inter-individual variability and are difficult to specify a priori, we additionally incorporated the actual number of MW episodes in each intentionality condition. This ensured that the power estimates were sensitive to the empirical distribution of event counts across content categories, which is particularly important for low-frequency types such as negative past-oriented or positive future-oriented MW.

Although these post-hoc estimates do not account for the full multilevel structure of the logistic models, they provide a reasonable approximation of the sensitivity of the observed effects. The resulting power indices were used to contextualize both significant and non-significant findings in light of the observed variability in MW occurrence rates.

These supplementary power estimates were used to evaluate the robustness of both significant and non-significant effects. Power exceeded .99 for all significant findings, including negative past-oriented, positive future-oriented, and negative future-oriented content. In contrast, non-significant categories such as neutral future-oriented and positive past-oriented content showed substantially lower power (approximately .06 and .30, respectively), indicating that their null results should be interpreted with caution.

**Reference**

Gelman A, Carlin J. Beyond Power Calculations: Assessing Type S (Sign) and Type M (Magnitude) Errors. Perspect Psychol Sci. 2014 Nov;9(6):641-51. doi: 10.1177/1745691614551642. PMID: 26186114.

**Table1. *Post-hoc Power of Differences in Odds Ratio of Content Between Intentional MW and Unintentional MW as Estimated by Multi-Level Logistic Regression***

| Content | Post-hoc Power |
| --- | --- |
| Future | **1.00** |
| Positive | **0.99** |
| Neutral | **0.99** |
| Negative | **0.26** |
| Positive past-oriented | **0.30** |
| Neutral past-oriented | **1.00** |
| Negative past-oriented | **1.00** |
| Positive future-oriented | **1.00** |
| Neutral future-oriented | **0.06** |
| Negative future-oriented | **1.00** |

**Python Code for Post-hoc Power Analysis Based on NormalIndPower**

from statsmodels.stats.power import NormalIndPower

from math import log

# ---- Observed odds ratio ----

observed_or = 1.75 # ← Replace this with your estimated OR

# ---- Convert to log-odds (required for NormalIndPower) ----

effect_size = log(observed_or)

# ---- Empirical sample sizes ----

n_intentional = 95 # ← Insert your sample sizes here

n_unintentional = 239 # ← Insert your sample sizes here

# Ratio of group sizes (group2 / group1)

ratio = n_unintentional / n_intentional

# ---- Initialize the power analysis object ----

power_analysis = NormalIndPower()

# ---- Compute post-hoc power ----

power = power_analysis.power(effect_size=effect_size,

nobs1=n_intentional,

alpha=0.05,

ratio=ratio,

alternative='two-sided')

print("Observed OR:", observed_or)

print("Log-odds effect size:", round(effect_size, 3))

print("Post-hoc Power:", round(power, 3))

―――――――――――――――――――――――――――――――――――――――

**Task Instructions（English followed by Japanese）**

Participants viewed single digits (1–9) presented sequentially at the center of the screen.
They were instructed to press the space bar whenever a digit other than “3” appeared.
When the digit “3” appeared, participants were instructed to withhold any response and remain still.

参加者には，画面中央に逐次提示される数字（1〜9）を観察するよう求められました。
「3」以外の数字が提示された場合にはスペースキーを押すように教示されました。
一方，「3」が提示された場合にはスペースキーを押さず，そのまま待機するよう求められました。

Thought-sampling probes appeared intermittently during the task.
Participants reported their mental state immediately prior to the prob**e** by selecting one of the following options:

1. Focused on the task
2. Intentionally mind-wandering
3. Unintentionally mind-wandering

Definitions shown to participants were as follows:

- *Focused on the task*: attention directed toward task performance, including thoughts related to numbers, responses, or task performance.
- *Mind-wandering (MW)*: thoughts entirely unrelated to the task (e.g., dinner plans, social activities, upcoming exams).
- *Intentional MW*: deliberately thinking about task-unrelated matters.
- *Unintentional MW*: attention drifting away despite the intention to stay focused.

課題の途中で複数回，思考プローブが提示されました。
参加者は，プローブ表示直前の自身の状態について，以下のいずれかを選択しました。

1. 課題に集中していた
2. 意図的にマインドワンダリングをしていた
3. 非意図的にマインドワンダリングをしていた

参加者には以下の定義が提示されました。

- *課題に集中していた*：課題遂行に注意が向いており，数字や反応，課題成績などの課題関連の思考を含む。
- *マインドワンダリング（MW）*：課題と完全に無関係な内容を考えている状態（例：夕食の予定，友人との計画，将来の試験など）。
- *意図的 MW*：自身の意思で課題無関係なことを考える状態。
- *非意図的 MW*：課題に集中しようとしても注意がそれてしまう状態。

They indicated the temporal orientation of its content using the following response options:

1. Past-related
2. Future-related
3. Not mind wandering

参加者は**プローブ直前に考えていた内容**の時間的志向性を以下から選択しました。

1. 過去に関連する
2. 未来に関連する
3. マインドワンダリングしていなかった

They rated the **emotional valence of their thought content**:

1. **Positive**
2. **Neutral**
3. **Negative**
4. **Not mind-wandering**

参加者は**プローブ直前に考えていた内容の感情価**を以下から選択しました。

1. **ポジティブ**
2. **ニュートラル**
3. **ネガティブ**
4. **マインドワンダリングしていなかった**

After each probe response, the task resumed automatically.
Participants were instructed to ask the experimenter if they required clarification.

各プローブへの回答後，課題は自動的に再開しました。
不明な点があれば実験者に質問するよう案内しました。
